# The Shortage in the Naval Medical Service

**Published:** 1914-07-18

**Authors:** 


### The Shortage in the Naval Medical Service.

For many years up to a decade or so ago, the Indian Medical Service presented attractions for capable young doctors far surpassing those offered by the other two homologous services—those of the Navy and of the British Army. In consequence the Indian Medical Service secured keen competition for its vacancies and an extremely high level of professional ability was maintained among its officers. There came a time when the disadvantages of the British Army medical service became so pronounced as compared with those of the Indian Service that a comprehensive revision of the conditions was imperative. When the terms of this revision were published, shrewd judges immediately perceived that the British Army Service would in the end attract a larger number of the best type of candidate than the Indian, and the prophecies then made have now for some time been amply fulfilled. It is a fact that the Royal Army Medical Corps offers advantages, professionally and materially, which cannot be obtained either in the Indian or in the Naval Service.

The last-named is, in fact, very seriously below its establishment for want of sufficient candidates; and the Council of the British Medical Association has approved a report upon this state of affairs which has been forwarded to the Admiralty. One reason of the loss of popularity of the Naval Medical Service is the changed distribution of the fleets. Naval life to-day, as the report points out, is largely a monotonous existence round outlandish parts of the home coasts. The former pleasant alternations of home and foreign stations with ever varying social and sporting facilities have gone for the vast majority.

The position of the married naval surgeon is also one of hardship and discomfort. To live in a mess aboard ship and to maintain also a home ashore, rarely seen by the way, involves a tax upon the married surgeon's resources which can hardly be met unless there are private means to help; in very few cases does such a help exist. By irksome regulations the disabilities of the Naval Medical Service have been multiplied, while many of the old attractions have disappeared. That this is so is shown not only by the numerous letters of complaint which the medical secretary of the British Medical Association has received, but also by the logic of facts. The Navy is about 70 medical officers short of its small establishment of ten years ago, although since then the *personnel* of the fleet has increased by about 21,000 men, and is still growing. The exact numbers are not known, it is stated, but it is reckoned that at least 80 to 100 ships must be short of their proper complement of surgeons. The reserves of officers are also declared to be of the most meagre description. It is to be hoped that the Admiralty will consider very seriously the unfortunate state of affairs disclosed in this report, and that something will be done to remedy the unsatisfactory shortage in this very important and very honourable service.

